# Current and Future Roles of Glycoprotein IIb–IIIa Inhibitors in Primary Angioplasty for ST-Segment Elevation Myocardial Infarction

**DOI:** 10.3390/biomedicines12092023

**Published:** 2024-09-04

**Authors:** Giuseppe De Luca, Ashley Verburg, Arnoud van’t Hof, Jurrien ten Berg, Dean J. Kereiakes, Barry S. Coller, Charles Michael Gibson

**Affiliations:** 1 Division of Cardiology, Polyclinic G. Martino, University of Messina, 98122 Messina, Italy; 2 Division of Cardiology, IRCSS Hospital Nuovo-Galeazzi Sant’Ambrogio, 20157 Milan, Italy; 3 Department of Cardiology, St. Antonius Hospital, 3435 CM Nieuwegein, The Netherlands; as.verburg@antoniusziekenhuis.nl (A.V.);; 4 Department of Cardiology, Maastricht University Medical Centre, 6229 HX Maastricht, The Netherlands; arnoud.vant.hof@mumc.nl; 5 Cardiovascular Research Institute Maastricht, 6229 ER Maastricht, The Netherlands; 6 The Carl and Edyth Lindner Research Center, The Christ Hospital, Cincinnati, OH 45219, USA; 7 Laboratory of Blood and Vascular Biology, Rockefeller University, New York, NY 10065, USA; collerb@rockefeller.edu; 8 Perfuse Study Group, Beth Israel Deaconess Medical Center, Harvard Medical School, Boston, MA 02114, USA

**Keywords:** GP IIb/IIIa inhibitors, primary angioplasty, STEMI

## Abstract

Acute myocardial infarction still represents the major cause of mortality in high-income countries. Therefore, considerable efforts have been focused on the treatment of myocardial infarctions in the acute and long-term phase, with special attention being paid to reperfusion strategies and adjunctive antithrombotic therapies. In fact, despite the successful mechanical recanalization of the epicardial conduit, a substantial percentage of patients still experience poor myocardial reperfusion or acute/subacute in-stent thrombosis. Due the delayed onset of action of currently available oral antiplatelet therapies, glycoprotein (GP) IIb–IIIa inhibitors could be expected to improve clinical outcomes, especially when administrated in the early phase of the infarction, due to the larger platelet composition of fresh thrombi, the dynamic nature of early thrombi, and the larger amount of viable myocardium existing in the early, as compared to a delayed, phase. Considerable evidence has accumulated regarding the benefits from GP IIb–IIIa inhibitors on mortality, especially among high-risk patients and when administered as an upstream strategy. Therefore, based on currently available data, GP IIb–IIIa inhibitors can be considered when the drug can be administered within the first 3 h of symptom onset and among high-risk patients (e.g., those with advanced Killip class or an anterior myocardial infarction). Even though it is not universally accepted, in our opinion, this strategy should be implemented in a pre-hospital setting (in an ambulance) or as soon as possible when arriving at the hospital (at the Emergency Room or Coronary Care Unit, irrespective of whether they are in spoke or hub hospitals). A new, second-generation GP IIb–IIIa inhibitor (zalunfiban) appears to be highly suitable as a pre-hospital pharmacological facilitation strategy at the time of first medical contact due to its favourable features, including its simple subcutaneous administration, rapid onset of action (15 min), and limited time of action (with a half-life of ~1 h), which is likely to minimize the risk of bleeding. The ongoing CELEBRATE trial, including 2499 STEMI patients, may potentially provide compelling data to support the upstream treatment of STEMI patients undergoing mechanical reperfusion. In fact, although the current therapeutic target of increased rates of timely reperfusion has been achieved, the future goal in myocardial infarction treatment should be to achieve the most rapid reperfusion prior to primary percutaneous coronary intervention, thus further minimizing myocardial damage, or, in some cases, even preventing it completely, and improving survival.

## 1. Introduction

**Figure 1:**
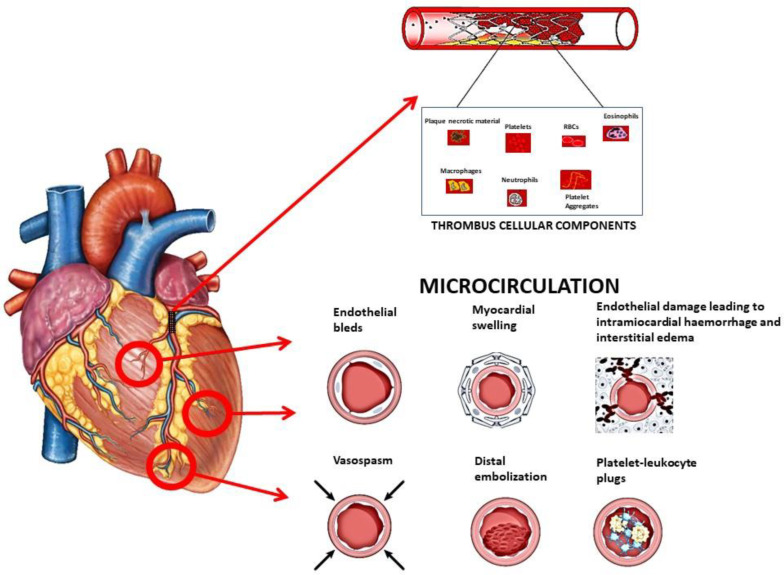
Thrombus cellular components and effects on coronary microcirculation.

ST-segment elevation myocardial infarction (STEMI) is the major cause of deaths in high-income countries [1]. Therefore, considerable efforts have been attempted by scientific societies and national governments in the last few decades to implement the organization of dedicated networks for the rapid treatment of STEMI in order to hasten coronary reperfusion in as many patients as possible. A plaque rupture and subsequent thrombus formation, in which platelets play a pivotal role, represent the main pathophysiologic drivers of acute coronary occlusion. In addition, platelets also mediate the impaired reperfusion of the microcirculation that may be observed despite the successful mechanical reopening of the occluded artery due to the distal embolization and release of inflammatory, prothrombotic, and vasospastic substances [2,3,4] (Figure 1). Several studies using invasive or imaging techniques have clearly demonstrated that TIMI 3 flow, mechanically achievable in the vast majority of patients, does not guarantee optimal reperfusion of the microcirculation, which remains suboptimal in about 20–40% of cases [5,6,7], with subsequent impaired short- and long-term outcomes.

Therefore, major efforts have increasingly focused on employing strong antithrombotic therapies to optimize mechanical reperfusion. The aim of the present article is to provide a current overview of the application of glycoprotein (GP) IIb–IIIa inhibitors, the most potent antiplatelet therapy, in the context of STEMI treated by Primary Percutaneous Coronary Intervention (PPCI).

## 2. Search Methodology

Electronic databases (MEDLINE and CENTRAL) and the scientific session abstracts in Circulation, the Journal of the American College of Cardiology, the European Heart Journal, and the American Journal of Cardiology, including presentations (TCT (www.tctmd.com), EuroPCR (www.europcr.com), ACC (www.acc.org), AHA (www.aha.org), and ESC (www.escardio.org)), were scanned from January 1990 to February 2024 (all assessed on 24 February 2024). We included the following listed keywords: GP IIb–IIIa inhibitors, abciximab, eptifibatide, tirofiban, zalunfiban, oral antiplatelet therapies, clopidogrel, prasugrel, ticagrelor, selatogrel, randomized trial, myocardial infarction, reperfusion, primary angioplasty, thrombolysis, facilitation, facilitated angioplasty, upstream, downstream, intracoronary, intravenous, unfractionated heparin, no-reflow phenomenon, inflammation, distal embolization, reinfarction, stent thrombosis, ischemia time, time to treatment, major bleeding, and bleeding complications. No language restrictions were enforced.

## 3. Molecular Insights, Pharmacodynamics, and Pharmacokinetics of GP IIB–IIIA Inhibitors

### 3.1. First-Generation Intravenous Drugs

#### 3.1.1. Abciximab

**Figure 2:**
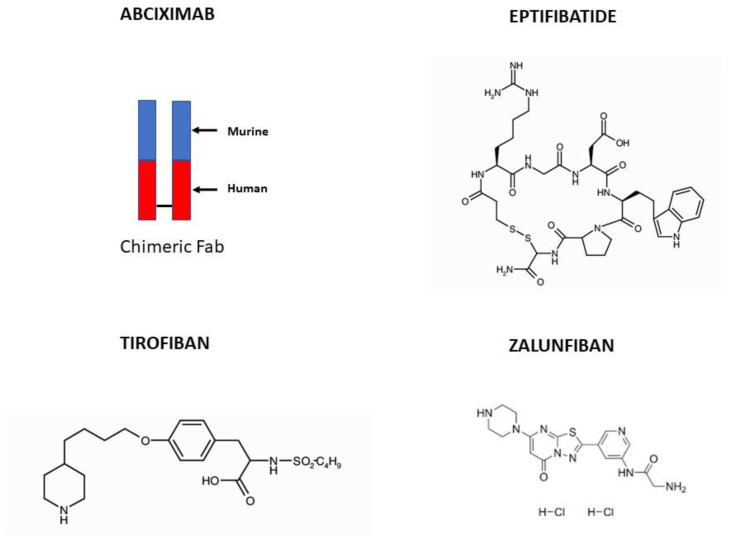
Structure of currently available intravenous GP IIb–IIIa inhibitors (abciximab, tirofiban, and eptifibatide) and the new-generation subcutaneous GP IIb–IIIa inhibitor zalunfiban.

**Figure 3:**
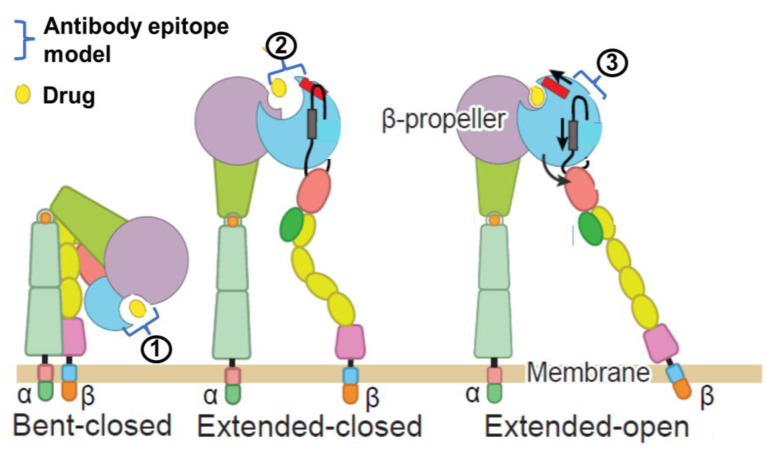
Conformations of GPIIb/IIIa and hypothetical models of small-molecule GPI-associated recruitments of an antibody to GPIIb/IIIa as a function of the conformational change induced by GPIs. GPIIb/IIIa is primarily in a bent–closed conformation (left) on inactivated platelets. With activation and/or ligand binding, GPIIb/IIIa undergoes extension of the headpiece from the legs (centre), and with ligand binding, it undergoes a swing-out motion that results in the adoption of a high-affinity extended–open conformation. The hypothetical models of small-molecule GPI-associated recruitments of an antibody to GPIIb/IIIa are the following: (1) The conformational changes (extension and swing-out) induced by the small molecules are neither sufficient nor necessary. This requires the antibody to be capable of binding to the drug αIIb and/or the β3 complex independent of the conformational change (2). The conformational change is necessary but not sufficient. This requires the epitope to require one or both of the conformational changes and the drug αIIb and/or the β3 complex (3. The conformational change is necessary and sufficient for antibody binding. This requires the antibody to bind to a neo-epitope exposed by one or both of the conformational changes, which is exclusively on αIIb and/or β3, rather than one that requires the drug αIIb and/or the β3 complex. If correct, zalunfiban, which does not induce conformational changes, should not induce thrombocytopenia caused by antibodies that bind according to models 2 and 3. The colors indicate different domains in αIIb and β 3. From top to bottom for each subunit. aIIb: Purple: Propeller; Green: Thigh; Orange: Knee; Light green: Calf 1 and 2; Orange: Transmembrane; Green: Cytoplasmic. β 3: Blue: βI; Pink insert in βI, β1-α1 loop; Orange: Hybrid; Green: PSI; Yellow: I-EGF1-4; Pink: β terminal; Blue: Transmembrane; Orange: Cytoplasmic. This figure was adapted from Lin et al., “A general chemical principle for creating closure-stabilizing integrin inhibitors”, Cell 185: 3533; 2022 [10], with permission from Elsevier, and Rikken et al., “Critical analysis of thrombocytopenia associated with glycoprotein IIb/IIIa inhibitors and potential role of zalunfiban, a novel small molecule glycoprotein inhibitor, in understanding the mechanism [s]”, J Am Heart Assoc 2023; 12: e031855 [11] with permission from Wiley.

Abciximab is a murine/human chimeric Fab fragment of the monoclonal 7E3 IgG3 antibody against GP IIb–IIIa receptors [8] (Figure 2). It is a competitive GP IIb–IIIa receptor inhibitor that rapidly, but reversibly, binds with high-affinity to the platelet receptor with a short plasma half-life (Table 1). The binding site is on the β3 chain of GP IIb–IIIa receptors [9] (Figure 3). While free plasma abciximab declines to low levels within minutes, the platelet-bound drug remains for up to one week, because it redistributes to newly produced platelets. These pharmacokinetic features support the safety of abciximab in patients with moderate to severe renal failure, including those on dialysis, without the need for dose adjustments. The recommended dosage of abciximab is a 0.25 mg/kg of intravenous bolus administered at least 10 min before the start of a PCI, followed by a continuous intravenous infusion of 0.125 μg/kg/min for 12 h.

#### 3.1.2. Eptifibatide

Eptifibatide is a cyclic heptapeptide containing a Lys-Gly-Asp (KGD) amino acid sequence (Figure 2), with high specificity for binding to GP IIb–IIIa receptors [12,13]. Eptifibatide binds to both the αIIb and β3 chains in the head region where they join, which is the binding site for fibrinogen, the von Willebrand factor, and the amino acid sequence Arg-Gly-Asp (RGD) (Figure 3). It is a selective, competitive GP IIb–IIIa receptor inhibitor and does not bind to other integrins. It rapidly associates with and dissociates from GP IIb–IIIa. It is eliminated by the kidney, being mainly excreted as an unchanged drug in the urine (Table 1). Two distinct loading or bolus dose regimens have been investigated (a 180 µg/kg IV bolus followed by a continuous infusion of 2 µg/kg/min for upstream use or as a 180 µg/kg IV bolus administered immediately before the initiation of a PCI followed by a continuous infusion of 2 µg/kg/min for up to 12–48 h and a second 180 µg/kg bolus 10 min after the first bolus when used downstream in the catheterization laboratory) [14,15]. In the case of renal dysfunction (with a creatinine clearance of <50 mL/min), the infusion should be reduced to 1 µg/kg/min. Eptifibatide is contraindicated in patients on dialysis.

#### 3.1.3. Tirofiban

Tirofiban is a nonpeptide tyrosine derivative (Figure 2). It binds to the same RGD binding site as eptifibatide [13,16] (Figure 3). It is a competitive, highly specific GP IIb–IIIa receptor antagonist and does not interact with other integrins. Tirofiban’s affinity for GP IIb–IIIa is higher than that of abciximab or eptifibatide. It is eliminated by the kidney. Tirofiban has a short biologic half-life, resulting in the recovery of platelet activity about 4 h following the cessation of therapy. Approximately 35% of tirofiban is unbound in the circulation, with a predominant renal clearance (65%); it can be eliminated with haemodialysis (Table 1). Two distinct loading or bolus dose regimens have been investigated—0.4 μg/kg/min infused over 30 min as upstream use in NSTEMI patients or 25 μg/kg over 3 min when used in the catheterization laboratory as downstream use or when administrated at the first medical contact before or during transportation to a catheterization laboratory (upstream use) in STEMI patients undergoing primary angioplasty [17,18]—and both are followed by a continuous infusion for 12–48 h. The dose is recommended to be reduced by 50% in patients with severe renal dysfunction (with a creatinine clearance of <30 mL/min).

### 3.2. Second-Generation Subcutaneous Drugs

#### Zalunfiban (RUC-4)

**Figure 4:**
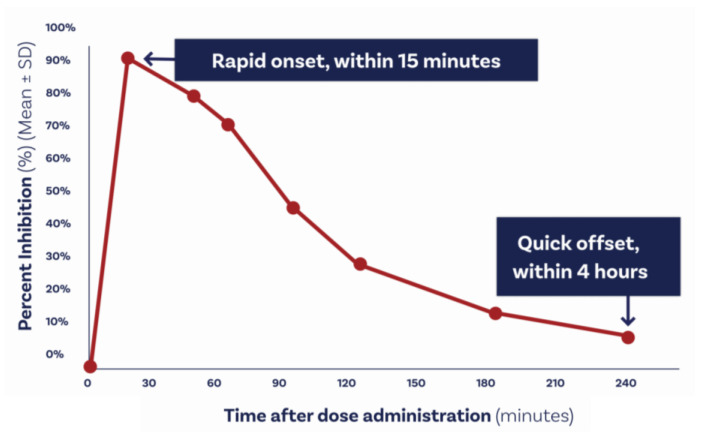
Inhibition of platelet aggregation by the subcutaneous administration of a new-generation GP IIb–IIIa inhibitor, zalunfiban, in patients with ST-segment elevation myocardial infarction. Adapted from Bor WL et al., EuroIntervention, 6 August 2021; 17 (5): e401–e410 [23].

Compared with eptifibatide and tirofiban, zalunfiban is a second-generation small-molecule platelet GP IIb–IIIa inhibitor (Figure 2) specifically designed to inhibit fibrinogen binding, platelet aggregation, and platelet thrombus formation without inducing the conformational changes in the receptor produced by the earlier I.V. drugs mentioned above [13], or fibrinogen [19], that result in the receptor adopting a high-affinity ligand binding state, with the exposure of otherwise-hidden epitopes on the receptor [20,21]. At the molecular level, zalunfiban binds to both the αIIb and β3 subunits of the receptor and displaces the Mg^2+^ ion from the metal ion-dependent adhesion site of the β3 subunit required for binding to fibrinogen and the von Willebrand factor; this locks the β3 subunit of the receptor in its inactive state without exposing neoepitopes [21] that are potential targets for preformed or treatment-induced antibodies that may contribute to the thrombocytopenia occasionally seen with eptifibatide or tirofiban [11,22] treatment. Zalunfiban is also designed to be biologically active following subcutaneous administration and highly soluble so that the anticipated maximal total human dose can be contained in less than 2 mL, opening the possibility of self-administration by an autoinjector. It has a rapid onset of action (reaching peak levels in ≤15 min) after subcutaneous administration, even in patients with STEMI [23], and achieves the high-grade inhibition of the platelet function in response to all agonists, including thrombin [24]. It has a limited time of action (~2 h with T_1/2_ = ~1 h), which may minimize the risk of bleeding (Figure 4).

## 4. Rationale for GP IIb–IIIa Inhibitors in STEMIs

### 4.1. Drug Resistance and Late Onset of Action of Oral Antiplatelet Therapies

Several factors clearly limit the role of oral antiplatelet therapies that inhibit the P2Y12 adenosine diphosphate (ADP) receptor in the early phase of myocardial infarctions. First and foremost, none of the P2Y12 antagonists inhibit high-dose thrombin-induced platelet aggregation [24,25]. Clopidogrel, being a prodrug with a complex metabolic pathway, has been shown to have a delayed onset of action and considerable interindividual variability in platelet inhibition, with up to 30% of individuals being classified as non-responders [26,27,28,29,30]. The variability in achieving effective platelet inhibition reflects several factors, including genetic polymorphisms of hepatic cytochrome enzymes involved in its activation, drug–drug interactions, variability in absorption, and other unidentified factors [31,32,33,34,35,36,37,38,39,40,41]. Interindividual variability and a delayed onset of action in the context of STEMI also applies to the newer and more potent P2Y12 inhibitors prasugrel and ticagrelor [42,43,44,45,46,47]. In particular, the Fabulous-Pro trial [42], which compared the antiplatelet effects of the bolus administration of prasugrel vs. tirofiban (with or without prasugrel) among STEMI patients undergoing a PPCI, showed that tirofiban, but not prasugrel, led to the optimal procedural platelet inhibition of an ADP-induced platelet aggregation. The delayed onset of action of oral antiplatelet therapies has been confirmed by the failure of both the upstream (in an ambulance) and downstream periprocedural administration of ticagrelor in STEMI patients to produce a reperfusion of the target coronary artery in patients undergoing a PPCI in the ATLANTIC trial [48]. Several factors may contribute to this delay, including the metabolic pathway of activation (prasugrel is also a prodrug); the administration of morphine, which reduces GI motility and drug absorption [49,50]; the gastric vasoconstriction that may follow the STEMI-associated rise in noradrenaline levels to counteract a severe acute left ventricular dysfunction and haemodynamic compromise [42,43,44,45,46,51,52].

Several new approaches have been investigated to hasten the absorption and the antiplatelet effects of oral therapies. A new ticagrelor formulation (orodisposable) [53] has been tested in the context of STEMI in the TASTER study, without any difference in residual platelet reactivity as compared to the standard formulation [54]. The crushed administration of new ADP antagonists has been shown to hasten the onset of action and the optimal inhibition of platelet aggregation [52,53,54,55]. In the COMPARE CRUSH study [55], which included STEMI patients undergoing PPCI, the pre-hospital administration of crushed prasugrel significantly enhanced the speed of onset of the inhibition of platelet aggregation. However, a substantial percentage of patients still exhibited suboptimal platelet inhibition when undergoing angiography (crushed, 43.3% vs. integral, 62.6%; *p* < 0.01). Similar results have been observed with ticagrelor [56,57,58].

Several other studies have investigated additional strategies to improve the absorption of oral antiplatelet agents with negative results [59]. The METAMORPHOSIS trial (Metoclopramide Administration as a Strategy to Overcome Morphine–ticagrelor Interaction in Patients with Unstable Angina Pectoris) [60] investigated the effects of a prokinetic agent (metoclopramide) to counterbalance the effects of opioids and demonstrated better plasma levels and platelet reactivity at 30 min after administration in patients with unstable angina. However, delayed absorption and high platelet reactivity may still persist despite using metoclopramide in STEMI patients [61]. Methylnaltrexone, a peripheral opioid–receptor antagonist, was investigated for its potential to antagonize delayed gastric emptying, but it showed no beneficial effects in terms of platelet reactivity [62]. The ON-TIME 3 (The Opioids and crushed Ticagrelor In Myocardial infarction Evaluation) trial investigated nonopioid analgesics (intravenous acetaminophen) to increase the bioavailability of oral P2Y12 inhibitors in STEMI patients and to reduce the likelihood that patients will still have high platelet reactivity [63] when undergoing a PPCI. No difference was observed in platelet reactivity compared with intravenous fentanyl. Similar results were observed in the PERSEUS trial [64]. All these data suggest the need for alternative agents to bridge the gap in platelet inhibition, such as GP IIb–IIIa inhibitors. In fact, the U.S. Food and Drug Administration (FDA) recommended considering parenteral antiplatelet therapy in patients with acute coronary syndrome requiring the coadministration of morphine or other opioid agonists.

### 4.2. Prognostic Implications of Ischemia Time and Early Reperfusion

The ischemia time is the major determinant of infarct sizes and survival in patients with STEMI undergoing either thrombolysis or mechanical reperfusion. Cannon et al. [65] analysed 27,000 STEMI patients in the U.S. National Registry of Myocardial Infarction (NRMI) 2 and found that the door-to-balloon time independently correlated with in-hospital mortality. A subsequent study, conducted by the Zwolle group, demonstrated, in a population of 1791 STEMI patients undergoing a primary PCI [66], that every 30 min of a delay to treatment was associated with a 7.5% increase in the adjusted relative risk of a 1-year mortality. Several additional studies demonstrated the prognostic value of the ischemia time in primary angioplasty [67,68,69,70,71]. The ischemia time also affects myocardial perfusion, as evaluated by the myocardial blush score, ST-segment resolution, enzymatic infarct size, and predischarge ejection fraction in patients who have a postprocedural TIMI 3 flow [72]. Thus, even when a PPCI can achieve a TIMI 3 flow, this advantage cannot overcome the deleterious impact of the ischemia time on myocardial necrosis and perfusion. In fact, the impact of the ischemia time on the infarct size has been confirmed in several investigations based on nuclear imaging techniques or cardiac MRIs [73,74,75,76,77,78,79,80].

Furthermore, the prognostic benefits of optimal preprocedural recanalization have been confirmed in several studies [81,82,83,84,85,86,87,88], especially in the early hours from symptom onsets and when long-distance transportation is needed. The rate of spontaneous recanalization (TIMI flow 2–3) has been described to range from 20 to 40%, with clear margins of improvement.

Similarly, the amount of myocardial salvage is significantly higher when recanalization occurs within the so-called golden hours, namely, the first three hours from symptom onset. The dedicated networks for a rapid STEMI treatment developed in the last few decades, in addition to public campaigns, were designed to hasten the presentation of STEMI patients and to increase the proportion presenting within the golden hours [89]. GP IIb–IIIa inhibitors have the potential to increase preprocedural reperfusion and, therefore, may play a pivotal role in STEMI networks to further shorten the ischemia time and increase the amount of myocardial salvage.

### 4.3. Time Dependency of Thrombus Composition

Animal and human studies have demonstrated the evolution of the thrombus composition over time from an initial plaque rupture and a coronary occlusion, with investigations documenting a higher content of platelets in the first few hours compared with an increasing presence of fibrin in later hours [90]. A subanalysis of the Horizons trial [91] demonstrated a clear time dependency of clinical benefits when GP IIb–IIIa inhibitors were administered compared to bivalirudin, with greater benefits being confined to the first 3 h from the onset of the symptoms of STEMI, a finding that was similar to that in the On-TIME 2 trial [92]. Similarly, the Early Glycoprotein IIb–IIIa Inhibitors in Primary Angioplasty (EGYPT) study demonstrated that a shorter time delay from symptom onset to upstream GP IIb–IIIa drug administration was associated with a more favourable outcome in regard to preprocedural TIMI flow 3, myocardial perfusion, distal embolization, and long-term survival as compared to a delayed administration [93]. These factors play in favour of the potential role of GP IIb–IIIa inhibitors in STEMI patients presenting in the first few hours of a symptom onset.

### 4.4. Incidence and Prognostic Implications of Distal Embolization

Several studies have demonstrated the role of distal embolization as a major determinant of the infarct size, poor reperfusion after a PPCI, and outcomes [94,95,96,97,98,99,100,101,102]. Sakuma et al. [94] showed, in animal models, that the presence of distal embolization is associated with increases in perfusion defects and the final infarct size. Several studies conducted on humans showed that distal embolization is a relatively common phenomenon during mechanical reperfusion for STEMI. Henriques et al. [95] observed an angiographically detectable distal embolization in approximately 16% of a STEMI population treated by PPCI, and it was associated with an impaired reperfusion, a larger infarct size, and a higher 5-year mortality. Similar observations were described by Napodano et al. for 400 STEMI patients treated by PPCI [96]. The EGYPT cooperative [97] showed the presence of distal embolization in up to 10% of its patients despite optimal antiplatelet therapy, and Fokkema et al. [98] found an occurrence of 6.3%. In a study by Yanuki et al., distal embolization occurred in 13.4% of patients despite the frequent use of thrombectomy [99].

Several clinical features have been associated with the increased risk of distal embolization [100], including advanced Killip class at presentation [101], diabetes [102,103,104,105], and an advanced age [106]. Several angiographic/procedural characteristics have also been associated with the increased risk of distal embolization, including a large vessel size, balloon predilatation [107], thrombus composition [108], large thrombus burden [109], and syntax score [110]. In contrast, neither gender [111] nor hypertension [112] are associated with an increased risk of distal embolization. Since thrombus burden is an important determinant of distal embolization [76,96,99,109,113,114], and GP IIb–IIIa inhibitors have the potential to reduce distal embolization.

### 4.5. No-Reflow Phenomenon

Inflammation, a spasm of the microcirculation, distal embolization, and mechanical compression have all been identified as important determinants of the “no-reflow” phenomenon. Several studies have demonstrated a neutrophil activation and accumulation in a damaged myocardium soon after the recanalization of an infarct-related artery [115,116]. The attenuation of neutrophil deformability after activation favours entrapment in the capillaries, leading to microvascular plugging. Leukocyte interactions with the endothelium and platelets, mediated, in part, by the glycoprotein adhesion molecules P-selectin, E-selectin, L-selectin, and intercellular adhesion molecule-1 (ICAM-1), are major contributors to the plugging. The adhesion of activated neutrophils to platelets involves both the platelet P-selectin and the neutrophil αMβ2, further solidifying the association between the thrombotic and inflammatory systems [11,19,20,21,22,23,24,25,26,27,28,29,30,31,32,33,34,35,36,37,38,39,40,41,42,43,44,45,46,47,48,49,50,51,52,53,54,55,56,57,58,59,60,61,62,63,64,65,66,67,68,69,70,71,72,73,74,75,76,77,78,79,80,81,82,83,84,85,86,87,88,89,90,91,92,93,94,95,96,97,98,99,100,101,102,103,104,105,106,107,108,109,110,111,112,113,114,115,116,117]. The complement cascade may further modulate these interactions [118]. Several studies have demonstrated, in vitro and in vivo, that GP III-IIIa inhibitors may attenuate inflammation [119,120,121,122,123,124,125]. Platelets also affect the microvascular regulation of coronary blood flows [126,127], increasing vascular resistance by the release of constrictive, pro-adhesive, and pro-inflammatory factors. Due to the above considerations, GP IIb–IIIa inhibitors have the potential to reduce the no-reflow phenomenon.

### 4.6. Incidence and Prognostic Implications of Stent Thrombosis

**Figure 5:**
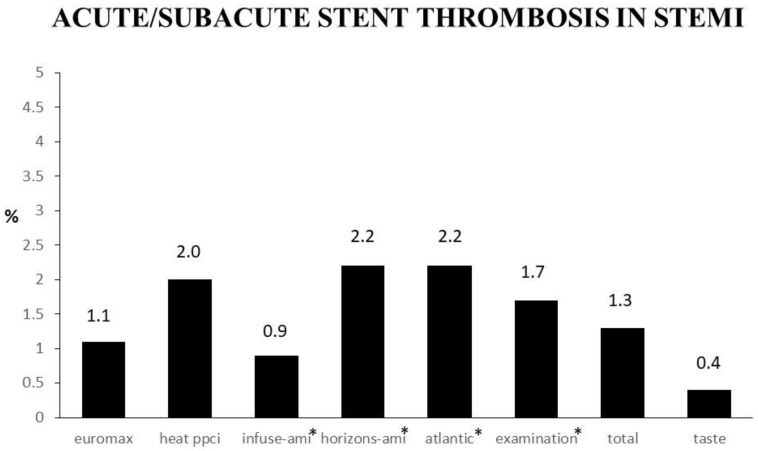
Bar graphs showing the rate of acute/subacute stent thrombosis in several trials on STEMI patients. * definite and probable stent thrombosis.

Despite the optimal recanalization of the infarct-related artery (IRA), reinfarction due to stent thrombosis may occur soon after the intervention or in a later phase, with a serious impact on survival [128,129,130]. Despite improved stent-implantation techniques [131], coronary stenting has not reduced reinfarction rates compared with balloon angioplasty [132,133,134]. The negative impact of stent thrombosis is supported by studies on more than 6000 patients (DESERT Cooperation) who were included in several randomized trials comparing first-generation drug-eluting stents to bare metal stents [135]. Generally, up to 50% of the events occurred within 1 month, with subacute stent thrombosis accounting for most of them. New-generation drug-eluting stents, due to their thinner struts and more biocompatible polymers or those with polymer-free technology, have minimized the risk of late and very late thrombotic events [136,137,138] but not the risk of stent thrombosis occurring within the early phase of an infarction, with rates of acute and subacute stent thrombosis being up to 2.2% (Figure 5), which may be prevented mainly by strong intravenous antiplatelet therapies.

### 4.7. Limitations of Unfractionated Heparin

Unfractionated heparin has the advantage of being low-cost, but it also has several disadvantages, including the following: (1) a dependency on antithrombin III for the inhibition of thrombin activity, (2) a sensitivity to platelet factor 4, (3) the inability to inhibit a clot-bound thrombin, (4) a marked inter-individual variability in therapeutic responses, (5) the need for frequent aPTT monitoring, (6) the ability to activate platelets, and (7) the risk of developing heparin-induced thrombocytopenia [139,140,141,142]. The adjunctive administration of GP IIb–IIIa inhibitors have the potential to counteract some of the above reported limitations.

## 5. Clinical Evidence

### 5.1. Periprocedural Intravenous Administration of GP IIb–IIIa Inhibitors

Among the GP IIb–IIIa inhibitors, abciximab is indubitably the most investigated drug with several randomized trials having been conducted in the context of STEMI. These studies showed significant benefits in myocardial perfusion [143,144] and distal microembolization [145,146]. Some large trials, such as CADILLAC (2082 patients) [147] and BRAVE-3 (800 patients) [148], showed no benefits in the infarct size or clinical outcome; they, however, also did not find a difference in major bleeding. The negative efficacy results may potentially be explained by the low-risk populations studied and the delayed time from symptom onset to intervention (with a median of 4.5 h in the BRAVE-3 trial) [148,149]. In trials without a strict patient-selection protocol in which higher-risk patients were enrolled, such as the one conducted by Antoniucci et al., abciximab provided significant benefits in death and reinfarction [150]. Several meta-analyses of randomized trials have been conducted [137,151,152] and have shown that the periprocedural administration of GP IIb–IIIa inhibitors is associated with a reduction in death and reinfarction [151,153] (Figure 3), with the benefits significantly being related to the patient-risk profile [137,152]. The use of GP IIb–IIIa inhibitors is associated with a significant increase in major bleeding (3.3% vs. 2.3%, *p* = 0.03), which is mainly due to data from the RAPPORT trial (16.6% vs. 9.5%, *p* = 0.02) [154]. However, in this trial, patients received a high-dose bolus of heparin [100 U/kg] and were treated with a femoral access approach, with most of the difference in bleeding being due to access-site bleeding (12.0 vs. 3.7%, *p* = 0.001).

Fewer data have been reported on the periprocedural administration of eptifibatide and tirofiban [155,156]. A meta-analysis [157] of six RCTs involving 708 tirofiban-treated patients and 721 control subjects showed significant beneficial effects from adjunctive periprocedural tirofiban administration in patients undergoing PPCI. The routine use of tirofiban decreased the rate of major adverse cardiovascular events (odds ratio (OR), 0.50; 95% confidence interval (CI), 0.26–0.94) and improved the corrected TIMI frame count (mean difference −8.48 (95% CI, from −12.62 to −4.34)), but there were no significant differences in the postprocedural TIMI flow grade 3, TIMI myocardial perfusion/blush grade 3, or mortality; importantly, the major bleeding, by TIMI criteria, was also not different. Concerning eptifibatide, only a few non-randomized studies have been reported [15]. A recent meta-analysis of studies in which patients also received oral P2Y12 antagonists also demonstrated a benefit of GP IIb–IIIa inhibitor therapy on a 30-day mortality (*p* = 0.01), 6-month mortality (*p* = 0.0001), and recurrent MI (*p* = 0.0006) [158]. In addition, a recent study from the U.K. comparing the results among interventional cardiologists who did or did not routinely use GP IIb–IIIa inhibitors demonstrated a mortality advantage for routine use, even when the more potent oral P2Y12 inhibitors are administered [159].

According to current European guidelines [160], GP IIb–IIIa inhibitors should be considered only for bailout therapy, a slow or no reflow, or a thrombotic complication (Class IIa). However, this recommendation is not evidence-based (level of evidence C).

Several randomized and non-randomized trials have compared small molecules vs. abciximab in primary angioplasty [155,160,161,162,163,164,165,166,167,168,169], and meta-analyses [170,171] of five tirofiban and one eptifibatide study showed similar outcomes in ischemic and bleeding complications between abciximab and the small molecules (Figure 4). Based on these data, current guidelines do not differentiate among the GP IIb–IIIa inhibitors given during PPCIs. 

Several randomized trials have been conducted to evaluate the relative effects of the intracoronary vs. intravenous administration of GP IIb–IIIa inhibitors. The INFUSE-AMI trial [172], including 452 STEMI patients with a large anterior MI treated within the first 4 h from the onset of symptoms, showed a significant reduction in infarct size by intracoronary abciximab administered locally at the site of the infarct lesion via the ClearWay RX Local Therapeutic Infusion Catheter as compared to intravenous infusion, without any benefits in the 30-day mortality (3.5% vs. 2.3%, *p* = NS) and acute/subacute stent thrombosis (0.9% vs. 0.9%, *p* = NS).

A meta-analysis of eight randomized trials [173], including 3259 STEMI patients, found that the intracoronary administration of abciximab was associated with a significant improvement in myocardial perfusion (OR (95% CI) = 1.76 (1.28–2.42), *p* < 0.001), but there was no benefit in mortality (OR (95% CI) = 0.85 (0.59–1.23), *p* = 0.39), reinfarction (OR (95% CI) = 0.79 (0.46–1.33), *p* = 0.37), or major bleeding complications (OR (95% CI) = 1.19 (0.76–1.87), *p* = 0.44). Therefore, the intracoronary administration of GP IIb–IIIa inhibitors does not appear to confer sufficient improvements in clinical outcomes so as to justify the delay required for its intracoronary administration in STEMI patients undergoing PPCIs.

### 5.2. Upstream Therapy

Whereas the ischemia time is a well-demonstrated major determinant of the infarct size, myocardial perfusion, and mortality among STEMI patients, attention has focused on achieving early pharmacological reperfusion, which may especially benefit high-risk patients and patients who require long-distance transportation and who present early [174,175]. As a result, several randomized trials have investigated the effects of upstream GP IIb–IIIa inhibitor administration in patients undergoing PPCI. The Early Glycoprotein IIb–IIIa inhibitors in Primary Angioplasty (EGYPT) cooperation was a study based on individual patients’ data from randomized trials [176], including 1662 patients, of which 840 patients (50.5%) were assigned to early and 822 patients (49.5%) to late GP IIb–IIIa inhibitor administration. This study showed that early upstream GP IIb–IIIa inhibitor use was associated with significant benefits in preprocedural and postprocedural TIMI flow, distal embolization, and myocardial perfusion and that these effects translated into benefits in mortality. Similar findings were observed in diabetic patients [88]. Most importantly, the benefits in mortality were maintained at up to 12 years of follow-up [177].

**Figure 6:**
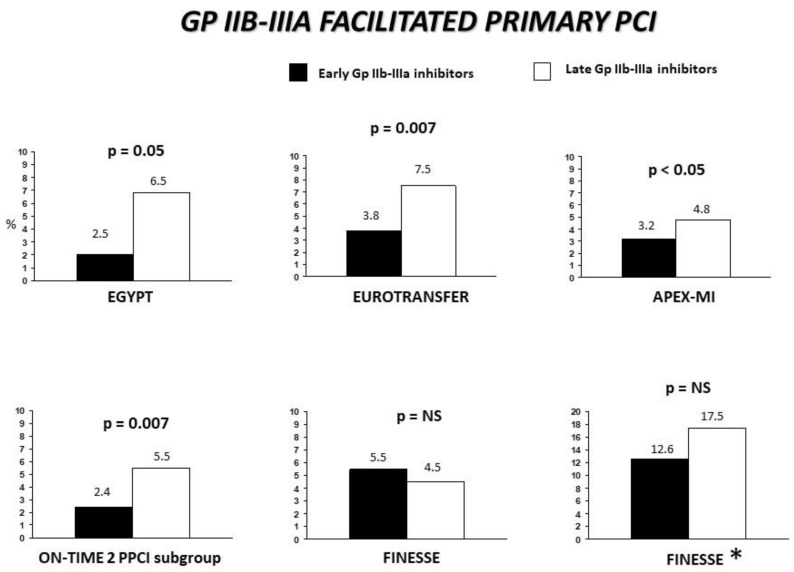
Mortality benefits of the administration of upstream GP IIb–IIIa inhibitors. * patients showing a TIMI risk score of >3, randomized at spoke centres (therefore undergoing transferring), and those treated with the study drug within 3 h of symptom onset.

The On-TIME 2 trial [178] was a double-blind, randomised, placebo-controlled study that included a total of 984 STEMI patients undergoing a PPCI who were randomly assigned to either receive an upstream pre-hospital (in-ambulance) high-bolus dose of tirofiban (n = 491) or a placebo (n = 493), in addition to aspirin (500 mg), unfractionated heparin (5000 IU), and clopidogrel (600 mg). The patients received the study drug at a median of 76 min after symptom onset and 55 min prior to the angiography/PCI. Tirofiban was associated with a significantly lower cumulative residual ST-segment deviation as compared to the placebo (10.9 ± 9.2 mm vs. 12.1± 9.4 mm; *p* = 0.028) at the time of arrival to the PCI centre as well as a benefit in the ST-segment resolution (*p* = 0.041 for trend). At 30 days, tirofiban was associated with improved outcomes based on the combined incidence of death, recurrent MI, urgent target vessel revascularization (TVR), or thrombotic bailout (26.0% vs. 32.9%, *p* = 0.020), without a significant difference in major bleeding (4% vs. 3%, *p* = 0.36). The pre-hospital administration of tirofiban was also associated with a significant reduction in acute stent thrombosis (0.2% vs. 3.0%, *p* < 0.001). In addition, a substudy of On-TIME 2 suggested that routine pre-treatment with a high-bolus dose of tirofiban was superior to provisional uses [179]. A pooled analysis, including 414 STEMI patients randomly assigned in the ambulance to receive either a high-bolus dose of tirofiban or no tirofiban (open-label phase), showed clear advantages with upstream pre-hospital tirofiban, with significant reductions in the 30-day MACE (5.8% vs. 8.6%, *p* = 0.043) and mortality (2.2% vs. 4.1%, *p* = 0.051), especially in those undergoing PPCI (2.4% vs. 5.5%, *p* = 0.007), without an increase in major bleeding (3.4% vs. 2.9%, *p* = 0.581). The benefits in mortality persisted at a 1-year follow-up (3.7% vs. 5.8%, *p* = 0.078) [180] (Figure 6). This pooled analysis also showed a higher number of “aborted” myocardial infarctions, that is, those without significant enzyme elevations in the patient group pre-treated with tirofiban early after symptom onset.

Several sets of registry data have additionally supported the beneficial effects of upstream GP IIb–IIIa inhibitors, in particular, the EUROTRANSFER Registry [181], the Emilia Romagna Registry [182], and data from the Assessment of Pexelizumab in Acute Myocardial Infarction (APEX-AMI) [183] [Figure 6].

So far, the FINESSE trial is the largest study conducted to investigate the benefits from a pharmacoinvasive approach [184], including up to 2500 STEMI patients who were randomly assigned, within 6 h of symptom onset, to the following treatments: the administration of (1) upstream half-dose reteplase plus abciximab, (2) upstream abciximab, or (3) downstream (periprocedural) abciximab. This study did not show any benefits of upstream abciximab in the preprocedural TIMI 2–3 flow (26 vs. 25%) or 90-day mortality (5.5 vs. 4.5%), and no difference was observed in major bleeding complications (4.1 vs. 2.6%, *p* = 0.13). Several aspects of this study merit consideration in interpreting the results. The study was prematurely stopped after 4 years due to slow recruitment. This contrasts with all of the trials that obtained positive results, which were conducted in highly experienced centres with fast recruitment rates. This difference may have been due to a potential selection bias. In addition, more than 50% of the patients were enrolled at hub centres, and so the therapy was not consistently upstream. Furthermore, many patients received the therapy after the first 3 h from symptom onset. Overall, the ischemia time was much longer than that observed in the EGYPT cooperation trial and the On-TIME 2 trial. In fact, posthoc analyses that address these issues showed benefits in outcomes among high-risk patients treated within the first 3 h of symptom onset who were enrolled in the ambulance or at a spoke hospital and then transferred for primary angioplasty [185,186].

Similar findings were observed in the On-TIME 2 trial, where early presenters experienced greater benefits from the upstream initiation of high-dose tirofiban [92,187]. The difference in the thrombus composition at various time intervals of symptoms may be a key factor in reconciling the variations observed in different studies. Silvain et al. [90] analysed the thrombus aspirates from STEMI patients undergoing PPCIs and found that the thrombi differed in composition with an increasing ischemia time, with the platelets being more evident within the first 3 h (freshly formed thrombi) and with an increasing amount of fibrin in the later phase. These findings have been confirmed in additional studies [188,189].

Although platelet dynamics have not been directly measured in STEMI patients, data from flow probe studies in animal models of STEMI and intravital microscopy studies in platelet and fibrin depositions after an acute vascular injury suggest that newly developing thrombi are highly dynamic, with platelet deposition and embolization preceding complete occlusion [190]. Abciximab is very effective in preventing platelet thrombi from forming as well as stopping the reaccumulation of platelets after embolization in both of these models, and zalunfiban was as effective as abciximab in a transgenic mouse model of a human platelet deposition after a vascular injury [21,191,192]. Studies have also demonstrated that under certain conditions, abciximab can not only stop further platelet aggregation when added to platelets that are in the midst of aggregating but can actually rapidly reverse the aggregation, as judged by a decrease in light transmission [193]. Collectively, these data demonstrate that potent parenteral antiplatelet therapy with GP IIb–IIIa inhibitors provides clinically meaningful benefits when administered within the first few hours of symptom onset, and this comports with early thrombi being richer in platelets than late thrombi. Thus, the accumulated evidence from multiple RCTs, supported by animal model data, indicate a benefit of upstream GP IIb–IIIa administration in the first few hours after symptom onset. When considered in light of the negative results of the ATLANTIC study [48], in which patients were administered ticagrelor in the pre-hospital phase, this indicates that the antiplatelet effects of oral P2Y12 inhibitors are inadequate for achieving benefits, emphasizing the need for the most potent antiplatelet agents, GP IIb–IIIa inhibitors. P2Y12 inhibitors are effective in reducing an ADP-induced platelet activation, but there are multiple other activators at the site of a thrombus, most notably the powerful activator thrombin.

Pre-hospital administration is much easier with a subcutaneous injection as with zalunfiban compared to the currently approved GP IIb–IIIa inhibitors and the P2Y12 inhibitor cangrelor, which need intravenous administration as a bolus plus a continuous infusion controlled by an electronic pump.

Based on the evidence that “time is muscle” and the positive effects of dedicated regional STEMI networks on reducing treatment delays [194,195,196], current guidelines strongly recommend establishing these STEMI networks to decrease system delays and increase the percentage of patients receiving reperfusion therapy—either pharmacological or mechanical—within the golden hours [186]. The large STEMI campaign launched by the “Stent for Life” initiative (www.stentforlife.com) [197] has strongly supported STEMI networks in Europe as well as in several other continents. Only GP IIb–IIIa inhibitors block the platelet aggregation induced by all of these agents, including thrombin, because it works downstream of all of the activators at the final common pathway. Selatogrel [198] is a new P2Y12 inhibitor that rapidly inhibits an ADP-induced platelet aggregation after subcutaneous administration, but it fails to inhibit a platelet aggregation initiated by the PAR1 thrombin receptor on platelets [69]. It is in advanced clinical development for self-administration by patients after the onset of chest pain [199].

Zalunfiban has a rapid onset of action (≤15 min) after subcutaneous administration, even in patients with STEMI, and achieves the high-grade inhibition of platelet function in response to agonists that stimulate both platelet ADP and thrombin receptors [23]. It also has a short duration of action (~2 h) to minimize the risk of bleeding. Furthermore, because it locks the receptor in its inactive conformation, it may be less likely to induce thrombocytopenia than the current GP IIb–IIIa inhibitors.

A very small (n = 27) post hoc analysis of a zalunfiban phase 2A study demonstrated a direct association of the dose administered and preprocedural TIMI flow and myocardial reperfusion grade, and an inverse relationship with thrombus grade [200]. Of note, in this study zalunfiban was only administered 10–15 min before the angiogram was performed.

The CELEBRATE trial (Celecor blinded randomized trial in ST-elevation myocardial infarction—NCT04825743) [201] was originally designed as a phase 2b trial to assess the restoration of the coronary artery blood flow at initial angiography and the resolution of ST-segment deviation 1 h after a PCI in STEMI patients randomly assigned in the ambulance to zalunfiban or a placebo. After a discussion with regulatory agencies, the steering committee modified the primary endpoint to a 30-day follow-up composite of a ranked, 7-point clinical- and troponin-level scale to assess the safety and efficacy of a single subcutaneous injection of zalunfiban in patients with STEMI who were anticipated to undergo a PPCI. Following modifications of the primary endpoints, the trial changed into a phase 3 clinical trial. This trial included 2499 STEMI patients who were enrolled within 4 h of symptom onset and who were randomized to a single subcutaneous injection containing zalunfiban at 0.110 mg/kg, zalunfiban at 0.130 mg/kg, or a placebo. Initially, this study was focused on pre-hospital ambulance-based therapy but was later expanded to include emergency department administration based on the blood flow data in the phase 2A study, suggesting a potential benefit on the blood flow within 10–15 min, the time between administration, and angiography.

The primary safety endpoint is bleeding events, according to the severe or life-threatening criterion of the Global Use of Strategies to Open Occluded Coronary Arteries (GUSTOs). Grades 3C and 5 of the Bleeding Academic Research Consortium (BARC) will also be reported in the future. CELEBRATE is one of the largest studies conducted so far on upstream pharmacological facilitation and will test this strategy only in patients presenting within the first 4 h after symptom onset in the modern era in which radial artery access predominates.

## 6. Safety Issues

### 6.1. Risk of Bleeding

**Figure 7:**
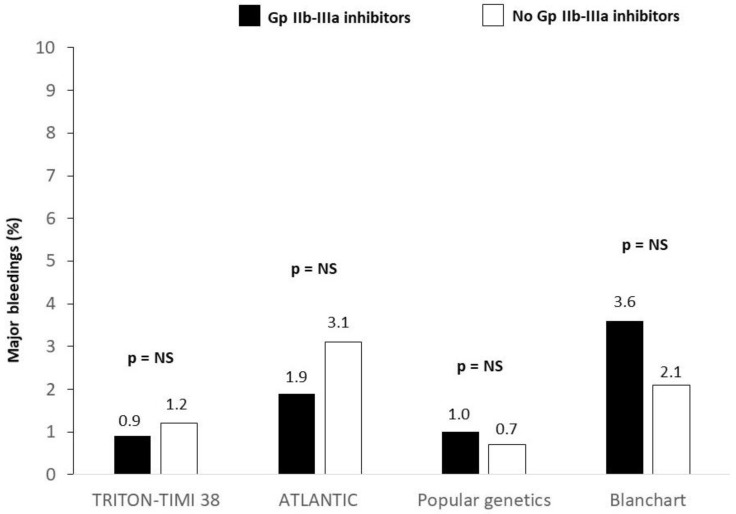
GP IIb–IIIa inhibitors and major bleeding complications in the modern era of new ADP antagonists.

GP IIb–IIIa inhibitors were initially associated with a substantial increase in the risks of bleeding complications, and the later addition of oral P2Y12 inhibitors heightened the concern about bleeding. However, data from TRITON-TIMI 38 [202], despite the prevalent use of a femoral artery approach, showed that the use of GP IIb–IIIa inhibitors was associated with a similar risk of major bleeding (1.2% vs. 0.9%) and intracranaial bleeding (0.1% vs. 0%) (Figure 7). Similar findings were observed in the ATLANTIC study [203], in which ticagrelor was studied, where the routine use of GP IIb–IIIa inhibitors did not increase the risk of bleeding complications. Most importantly, the switch to a radial artery approach instead of a femoral artery approach significantly reduced the risk of access-site bleeding [204]. A subanalysis of the Popular Genetics trial, in which 90% of 2378 patients were treated with ticagrelor and 85% with a radial approach, showed that GP IIb–IIIa administration was associated with fewer thrombotic events (hazard ratio (HR), 0.22; 95% confidence interval (CI), 0.09–0.55); fewer MIs (HR, 0.24; 95% CI, 0.08–0.73) and an increase in minor bleedings (HR, 2.32; 95% CI, 1.43–3.76), but no difference in major bleeding (HR, 0.69; 95% CI, 0.19–2.57) was found [205]. An Italian registry conducted between 2015 and 2018, including 472 STEMI patients undergoing PPCI who were treated with prasugrel or ticagrelor and a periprocedural high-dose bolus of tirofiban, showed a low rate of BARC (2.1%) and 3–5 BARC (1%) bleeding despite the bolus being followed for up to 18 h by a tirofiban infusion in 65% of the population [206]. A recent meta-regression analysis showed that the mortality benefit of the radial artery approach was significantly modulated by the use of GP IIb–IIIa inhibitors [207]. In fact, the greater benefit of a radial access was observed in studies with a greater use of these drugs. A recent meta-analysis of studies of GP IIb–IIIa inhibitors in STEMIs [158], in which the data were separated into studies reported in 1998–2007 vs. 2008–2016, found that the major bleeding rates were 5.8 vs. 4.3% (*p* = 0.04) among those treated with or without GP IIb–IIIa inhibitors, respectively, in the early time period and 3.5 vs. 2.4% (*p* = 0.05) the later time period. Thus, even with the introduction of the more potent P2Y12 oral agents, the major bleeding rate in the later period was actually lower that in the control group in the early period.

It is likely that a postprocedural infusion of a GP IIb–IIIa inhibitor is no longer needed in view of the use of the new, more potent ADP antagonists and the near-universal use of the radial artery approach. The new subcutaneous GP IIb–IIIa inhibitor zalunfiban was specifically designed to be used in combination with the newer oral P2Y12 inhibitors, since its rapid onset and short time of action complement the longer time it takes to reach the maximal antiplatelet effects of the oral agents while minimizing the risk of bleeding. The ongoing CELEBRATE trial [201] will provide additional data on this important issue.

### 6.2. Thrombocytopenia

Thrombocytopenia represents a rare but serious and potentially even fatal complication of the intravenous GP IIb–IIIa inhibitors. Marked thrombocytopenia (<20,000/μL) occurs in less than 1% of patients treated for the first time [11]. A meta-analysis of 29 randomized studies, including 123,419 patients treated with the three intravenous agents (abciximab, tirofiban, and eptifibatide) and four oral agents that were never approved for clinical use, found mild thrombocytopenia (<100,000 platelets/μL) in 3.3% of the treated patients vs. 2.2% of the patients that received a placebo (relative risk (RR), 1.63 (95% CI, 1.48–1.79)) and severe thrombocytopenia (<50,000 platelets/μL) in 0.8% vs. 0.2% of the patients (RR, 3.51; 95% CI, 2.68–4.58) [208]. In the analysis restricted to intravenous GP IIb–IIIa inhibitors, the occurrence of thrombocytopenia was higher with abciximab (RR = 2.93) and tirofiban (RR = 2.79) but not eptifibatide (RR = 1.05). Abciximab-associated thrombocytopenia can be due to either preformed antibodies (immediate) or antibodies induced in response to abciximab (delayed). Small-molecule GP IIb–IIIa inhibitors bind αIIbβ3, like natural ligands, inducing a major conformational change of the receptor that potentially creates neoepitopes recognized by either preformed or induced antibodies to the drug–receptor complex. It is not clear whether the antibody binding depends on the conformational change and whether the drug contributes directly to the epitope. Zalunfiban does not induce the conformational changes, and, therefore, if the thrombocytopenia is caused, in part, by the conformational change, it may have a reduced risk of this complication.

## 7. Genetic Polymorphisms of GP IIB–IIIA Receptors and Response to GP IIB–IIIA Inhibitors

GPIIb/IIIa [integrin α_IIb_β_3_] is an integrin complex located on the surface of platelets. It is a transmembrane receptor for fibrinogen and the von Willebrand factor that contributes to platelet adhesion and aggregation. It is expressed at high density on the platelet surface, with ~100,000 copies per platelet. Platelet activation by major platelet stimuli leads to a major conformational change in the receptor that increases its affinity for its ligands [209,210].

The HPA-1a,b polymorphism of GPIIb/IIIa, previously termed Pl^A1^/Pl^A2^, consists of a Leu33Pro substitution and is well recognized as being associated with neonatal isoimmune thrombocytopenia [211]. Approximately 25% of the white population carries at least one HPA-1b allele [212]. Investigators have reported variable results with regard to the variant’s effects on platelet function, and similarly, there have been variable reports with regard to whether the variant affects the response to GP-IIb/IIIa inhibitors [213,214,215,216,217,218,219,220,221,222,223]. Contrasting results have also been reported on the association of this allelic variant with the increased risk of myocardial infarction, impaired myocardial reperfusion, in-stent restenosis, coronary artery disease, and stroke [224,225,226,227,228,229,230,231,232,233,234,235,236,237,238]. No data have been reported so far on the impact of polymorphisms on clinical benefits from the administration of GP IIb–IIIa inhibitors.

## 8. Conclusions

Despite successful coronary recanalization, a substantial proportion of STEMI patients still experience suboptimal myocardial perfusion and reocclusion. Therefore, studies in recent decades have focused on optimizing antithrombotic therapies, in particular adjunctive GP IIb–IIIa inhibitors, which have been shown to reduce such complications and which have a much faster onset of action than oral ADP antagonists. The benefits of a therapy with powerful antiplatelet agents are expected to be greater in the early phase of an infarction, when the thrombi are platelet-rich and more likely to still be dynamic, and when there is the greatest opportunity to avoid irreversible damage to a viable myocardium. Robust evidence has accumulated on the benefits of GP IIb–IIIa inhibitor therapy in preventing stent thrombosis and improving mortality, especially when high-risk patients are treated upstream. With modern technology and the radial artery access approach, GP IIb–IIIa inhibitors confer little, if any, increased risks of major bleeding, even when associated with new ADP antagonists.

Therefore, based on currently available data, GP IIb–IIIa inhibitors could be considered as an upstream strategy in those presenting within the first three hours of symptom onset and among high-risk patients, such as those with advanced Killip class or anterior MI. The use of the subcutaneous GP IIb–IIIa inhibitor zalunfiban in the CELEBRATE trial [201] should facilitate early administration and thus provide crucial feasibility, efficacy, and safety data regarding this approach. With the increasing incidence of STEMI in low- and middle-income countries, where rapid access to a PCI centre is limited, zalunfiban may also be a valuable adjunct to aspirin and P2Y12 inhibitors, if it improves reperfusion and limits the infarct size in a meaningful percentage of patients. While the identification of new risk factors and the implementation of primary and secondary preventive strategies remain crucial in minimizing the development of coronary artery diseases and STEMI [239,240,241,242], early diagnosis and rapid reperfusion with optimal antithrombotic and mechanical therapies [243,244] remain the key elements to reduce the short-term and long-term mortality of patients with STEMI.

## Figures and Tables

**Table 1:** Characteristics of GP IIb–IIIa inhibitors.

	Abciximab	Eptifibatide	Tirofiban	Zalunfiban
Type	Antibody Fab fragment	Cyclic peptide	Non-peptide	Non-peptide
Molecular weight (Da)	47,600	832	495	386
Inhibition	Noncompetitive	Competitive	Competitive	Competitive
Binding	Reversible	Reversible	Reversible	Reversible
Platelet affinity	High	Low	High	Intermediate
Plasma half-life	10–30 min	2.5 h	2 h	
Recovery of platelet function	Slow (24–48 h)	Fast (<4 h)	Fast (4–8 h)	Fastest (~2 h)
Antigenicity	Least rare	Rarest	Rare	Unknown
Clearance	platelet binding; unbound via unknown mechanisms	Renal (60–70%) or biliary (20–30%)	Renal (98%)	Renal clearance of inactive metabolites
Administration	Intravenous	Intravenous	Intravenous	Subcutaneous
Recommended dosage				
Bolus	0.25 μg/kg	181 μg/kg × 2	26 μg/kg	0.11 and 0.13 mg/kg under study
Infusion	0.125 μg/kg/min (12 h)	2 μg/kg/min	0.15 μg/kg/min	None
